# Independent Effects of Kidney Function and Cholesterol Efflux on Cardiovascular Mortality

**DOI:** 10.3390/biomedicines10081832

**Published:** 2022-07-29

**Authors:** Andreas Ritsch, Monika Hunjadi, Tatjana Stojakovic, Jürgen E. Scherberich, Günther Silbernagel, Hubert Scharnagl, Graciela E. Delgado, Marcus E. Kleber, Winfried März

**Affiliations:** 1 Department of Internal Medicine, Medical University of Innsbruck, 6020 Innsbruck, Austria; monika.hunjadi@alumni.i-med.ac.at; 2 Clinical Institute of Medical and Chemical Laboratory Diagnostics, University Hospital Graz, 8036 Graz, Austria; tatjana.stojakovic@uniklinikum.kages.at; 3 Department of Nephrology and Clinical Immunology, Klinikum Muenchen-Harlaching, Teaching Hospital of the Ludwig-Maximilians University, 81545 Munich, Germany; j.scherberich@web.de; 4 Division of Angiology, Department of Internal Medicine, Medical University of Graz, 8036 Graz, Austria; guenther.silbernagel@medunigraz.at; 5 Clinical Institute of Medical and Chemical Laboratory Diagnostics, Medical University of Graz, 8036 Graz, Austria; hubert.scharnagl@medunigraz.at (H.S.); winfried.maerz@synlab.com (W.M.); 6 Vth Department of Medicine, Medical Faculty Mannheim, Heidelberg University, 68167 Mannheim, Germany; graciela.delgado@medma.uni-heidelberg.de (G.E.D.); marcus.kleber@medma.uni-heidelberg.de (M.E.K.); 7 Synlab Academy, Synlab Holding Deutschland GmbH, 86156 Augsburg, Germany

**Keywords:** single nucleotide polymorphism, cardiovascular risk, cholesterol efflux capacity, high-density lipoprotein, kidney function, uromodulin

## Abstract

Background: Impaired renal function is associated with cardiovascular and all-cause mortality. In the general population, HDL-cholesterol is associated with cardiovascular events, which is not true in patients with chronic kidney disease (CKD). This has been attributed to abnormal HDL function in CKD. Methods: In this study, we analyzed the association of genetic markers for kidney function with cholesterol efflux capacity as one of the major HDL functions, as well as with cardiovascular mortality, in 2469 patients of the Ludwigshafen Risk and Cardiovascular Health Study who all underwent coronary angiography. Results: A genetic score of 53 SNPs associated with GRF and the uromodulin SNP rs12917707 were inversely correlated with cholesterol efflux capacity. This was in line with the observed association between cholesterol efflux capacity and kidney function in these patients. Adjustment for eGFR and uromodulin as markers of kidney function did not affect the relationship between cholesterol efflux and cardiovascular mortality. Conclusions: Our data propose the view that cholesterol efflux and kidney function are exerting their effects on cardiovascular mortality via different and independent pathways. Decreased cholesterol efflux may therefore not mediate the effects of impaired kidney function on cardiovascular mortality.

## 1. Introduction

Patients with chronic kidney disease (CKD) disclose a high burden for cardiovascular disease (CVD). Even mild kidney dysfunction increases the risk of cardiovascular disease [1]. In addition, a reduction in glomerular filtration rate has been identified as a potent and independent risk factor [2]. Low levels of high-density lipoprotein cholesterol (HDL-C) indicate a risk for atherosclerotic cardiovascular disease in the general population [3]. However, there is a growing body of evidence that raising HDL-C levels may not be atheroprotective throughout. Patients with genetically elevated apoA-I and HDL-C do not have a reduced risk for cardiovascular disease [4]. Intervention studies of CETP inhibitors or niacin (in combination with statins) have been stopped because they failed to show a beneficial effect on primary endpoints [5]. In addition, higher HDL-C levels were not associated with reduced mortality risk and CVD in patients with reduced kidney function [6]. These observations do not support a causal role of HDL-C in atherosclerosis and may suggest the presence of other actions of HDL particles not readily reflected by their cholesterol content. A key function of HDL among numerous actions proposed in the last few years is the ability to promote the efflux of cholesterol from peripheral cells and to initiate shuttling of cholesterol back to the liver. The reverse cholesterol transport is considered atheroprotective by transferring excess cholesterol from peripheral cells back to the liver where it is secreted into the bile or converted into bile acids. Cholesterol efflux from cholesterol-laden macrophages represents an initial step within this pathway and has been shown to prevent atherosclerosis in animal models [7]. The well-studied association between HDL-cholesterol and cardiovascular events is not seen in patients with chronic kidney disease (CKD), pointing to abnormal HDL function in these patients. This is in agreement with studies showing that HDL dysfunction, including reduced cholesterol efflux capacity, begins in early CKD and progresses with declining renal function [8,9]. We therefore decided to investigate the genetic background of reduced cholesterol efflux as well as the influence of kidney function on cholesterol efflux capacity and cardiovascular mortality in patients with a high prevalence of CKD. In addition to eGFR as a well-established parameter of kidney function, we extended our studies to uromodulin, which has been shown to improve risk prediction when added to established cardiovascular risk prediction scores [10].

## 2. Materials and Methods

### 2.1. Study Participants

We studied 2468 participants of the LUdwigshafen RIsk and Cardiovascular Health (LURIC) study [11]. 1032 patients (29%) of the original study population were not included in this study as not enough sample volume of serum required for cholesterol efflux measurement had been available from these patients. Inclusion criteria were: German ancestry, clinical stability except for acute coronary syndromes, and the availability of a coronary angiogram. The indications for angiography in individuals in clinically-stable condition were chest pain and/or non-invasive test results consistent with myocardial ischemia. Individuals suffering from acute illness other than acute coronary syndromes, chronic non-cardiac diseases, or malignancy within the past 5 years and subjects unable to understand the purpose of the study were excluded. The study was approved by the Ethics Committee at the “Aerztekammer Rheinland-Pfalz” and was performed in accordance with the declaration of Helsinki (837.255.97 [1394], approved 8 January 1999). Informed written consent was obtained from all participants. Coronary artery disease (CAD) was assessed by angiography with maximum luminal narrowing estimated by visual analysis. Clinically-relevant CAD was defined as the occurrence of ≥1 stenosis of 20% in ≥1 of 15 coronary segments. Individuals with stenoses <20% were considered as not having CAD. Diabetes mellitus was diagnosed when plasma glucose was >1.25 g/L in the fasting state or >2.00 g/L 2 h after an oral glucose load [12], or when antidiabetic medical treatment was prescribed. Hypertension was diagnosed when the systolic and/or diastolic blood pressure exceeded 140 and/or 90 mm Hg, respectively, or when a patient was on antihypertensive medication. Information on vitals status was obtained from local registries. Among the 2468 people studied, 717 deaths (29.0%) occurred during a median follow-up of 9.9 (8.5–10.7) years. Cardiovascular death included sudden death, fatal myocardial infarction, death due to congestive heart failure, death immediately after intervention to treat CAD, fatal stroke, and other causes of death due to CAD.

### 2.2. Laboratory Procedures

Fasting blood samples were collected prior to angiography. The standard laboratory methods have been described [11]. The estimated glomerular filtration rate (eGFR) was calculated using the 2012 CKD-EPI eGFRcreat-cys equation as previously described [6,13]. eGFR could not be calculated for five patients due to missing data for the completion of the respective formula. Serum uromodulin was measured using a sensitive ELISA specifically adapted to serum specimens [14].

### 2.3. Cholesterol Efflux Capacity

Cholesterol efflux capacity was quantified in blood samples as described [15]. Briefly, J774 cells, derived from a murine macrophage cell line, were plated and radiolabeled with 2 μCi of 3H-cholesterol per milliliter. Cells were incubated with 0.3 mM of cAMP (C3912, Sigma-Aldrich, Waltham, MA, USA) to upregulate ABCA1. Subsequently, efflux medium containing 2.8% apolipoprotein B–depleted serum was added for 4 h. All steps were performed in the presence of 2 µg per milliliter acyl–co-enzyme A:cholesterol acyltransferase inhibitor (Sc-215839A, Santa-Cruz Biotechnology, Dallas, TX, USA). Liquid scintillation counting was used to quantify the efflux of radioactive cholesterol from the cells. Percent efflux was calculated by the following formula: [(microcuries of 3H-cholesterol in medium containing 2.8% apolipoprotein B–depleted serum–microcuries of 3H-cholesterol in serum-free medium)/microcuries of 3H-cholesterol in cells extracted before the efflux step] × 100. To correct for inter-assay variation across plates, a pooled serum control was included on each plate. Values for serum samples from patients are given in percentage of this control (% C). All assays were performed in triplicate.

### 2.4. Genetic Analyses

Genotyping was performed using the Affymetrix Human SNP Array 6.0. Genotype imputation was performed using MACH 1.0 and HapMap II CEU (release 22, NCBI build 36, dbSNP 126) samples as a reference. After imputation, 2,543,887 SNPs were available. SNPs with a squared correlation of ≥0.3 between imputed and true genotypes were considered well imputed. A genetic score was calculated based on 53 independent SNPs related to eGFR [16]. For weighting, β estimates on eGFR values of all included SNPs were taken as described [17].

### 2.5. Statistical Analysis

Normally-distributed data are presented as mean ± SD. CETP, triglycerides, adiponectin, interleukin-6 (IL-6), and C-reactive protein (CRP) exhibited a skewed distribution and are presented as a median and interquartile (Q1, Q3) range. Parameters that are not normally-distributed were transformed logarithmically for statistical analyses. The χ2 test and analysis of variance were used to compare the distributions of the variables across the eGFR categories. The effects of cardiovascular risk factors, CAD-status, and intake of lipid-lowering drugs on cholesterol efflux levels were determined using general linear models using cholesterol efflux as the dependent variable and age, lipid lowering therapy, CAD-status, body mass index (BMI), diabetes mellitus, smoking history (never, former, current), LDL-C/HDL-C ratio, and triglycerides as independent variables. Multivariate adjustments were performed for age, gender, intake of statins, CAD-status [none, stable CAD, unstable CAD, non-ST-elevation myocardial infarction (NSTEMI), or ST-elevation myocardial infarction (STEMI)], BMI, smoking status, LDL-C/HDL-C ratio, triglycerides, and metabolic syndrome/type 2 diabetes mellitus. The effects of eGFR and cholesterol efflux capacity on the SNP-score corresponding to the sum of eGFR-increasing alleles were determined using general linear models using the SNP-score as the dependent variable and age, gender, lipid-lowering therapy, CAD-status, body mass index (BMI), diabetes mellitus, smoking history (never, former, current), hypertension, LDL-C/HDL-C ratio, apolipoprotein AI, and triglycerides as independent variables. Cox proportional hazard models were used to examine the effect of cholesterol efflux capacity on mortality. A multivariable adjustment was performed for the intake of lipid-lowering drugs, age, gender, CAD, BMI, diabetes mellitus, smoking, LDL-C/HDL-C ratio, and triglycerides. Moreover, the interaction term between cholesterol efflux and eGFR as well as uromodulin with regard to cardiovascular mortality was studied, including the respective interaction terms as co-variates. Additionally, residuals from linear regression models of eGFR as well as uromodulin on cholesterol efflux capacity were used instead of efflux capacity in the Cox proportional hazard models to test for a possible interplay between parameters of kidney function and cholesterol efflux capacity. All statistical tests were 2-sided; *p* < 0.05 was considered significant. The SPSS 22.0 statistical package (SPSS Inc., Chicago, IL, USA) was used.

## 3. Results

### 3.1. Study Participants

Serum samples from 2468 individuals from the LURIC study were available for measurement of cholesterol efflux capacity. Clinical and biochemical characteristics of the study population were classified into three categories by their estimated GFR, as shown in Table 1. Patients with moderate to severe impaired kidney function (eGFR < 60 mL/min per 1.73 m^2^) displayed typical features of uremic dyslipidemia, including lower LDL cholesterol (LDL-C) and HDL-C, and higher triglycerides. This was accompanied by decreased concentrations of ApoA-I and ApoA-II, but unchanged ApoB. Low HDL-C as well as increased triglycerides levels are well in line with the increased levels of CETP in patients with low eGFR. No differences in the use of lipid-lowering drugs were observed, but the rates of diabetes mellitus and patients of the female sex were higher in patients with severe kidney failure (Table 1). During a median follow-up time of 9.9 years, 717 participants (29.2%) died—62.5 % (*n* = 448) of all deaths were due to cardiovascular causes.

### 3.2. Cholesterol Efflux Capacity and Kidney Function

**Figure 1:**
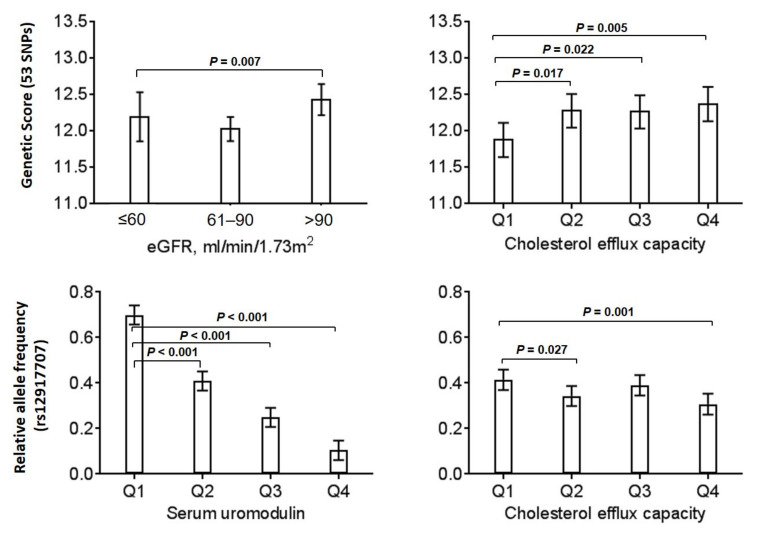
Association of kidney function parameters with corresponding genetic data from SNP analysis. Upper panel: Association of a genetic score of 53 independent SNPs with eGFR (**left**) and with cholesterol efflux capacity (**right**). Lower panel: Association of polymorphism rs12917707 with serum uromodulin (**left**) and with cholesterol efflux capacity (**right**). Diagrams are showing estimated marginal means and 95% confidence intervals obtained in a general linear model, adjusted for age, gender, lipid-lowering therapy, CAD-status, body mass index (BMI), diabetes mellitus, smoking history (never, former, current), hypertension, LDL-C/HDL-C ratio, apolipoprotein AI, and triglycerides.

In a recent genome-wide association study combining data from 133,413 individuals with replication in 42,166 individuals, 53 loci associated with eGFR were found [16]. Identified genes were enriched for expression in kidney tissues and in pathways relevant to kidney development, structure, and function. In the current study, a genetic score based on all 53 SNPs was higher in control patients when compared to patients with moderate kidney insufficiency and lower in patients of the lowest cholesterol efflux quartile (Figure 1, upper panel). This is supported by parallel investigations of the association of the uromodulin-related polymorphism, rs12917707, with serum uromodulin and the cholesterol efflux capacity, respectively (Figure 1, lower panel).

**Figure 2:**
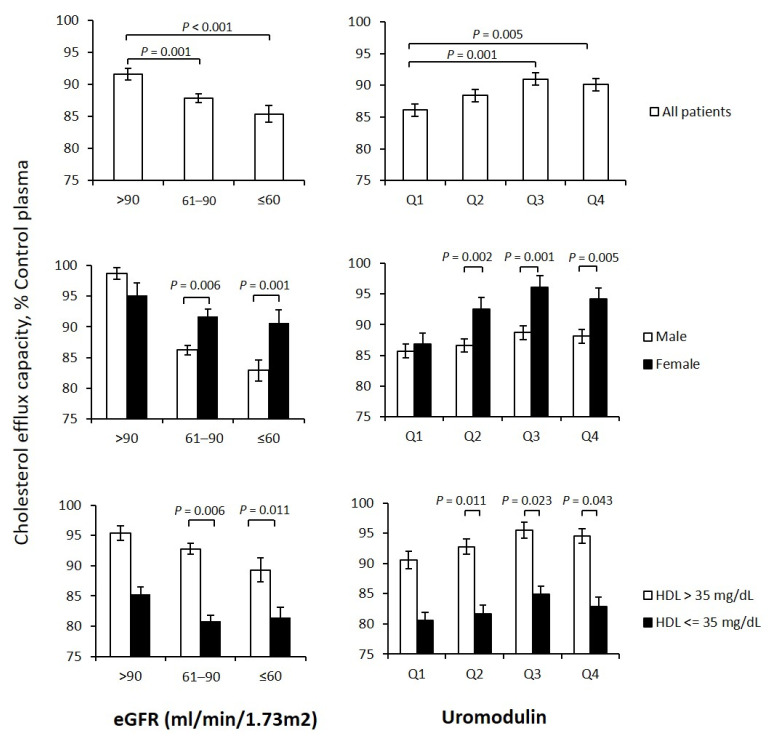
Association of kidney function parameters with cholesterol efflux. Multivariable-adjusted estimated marginal means of cholesterol efflux capacity in subgroups of participants of the LURIC study according to estimated glomerular filtration rate (eGFR, **left** panel) as well as uromodulin plasma concentration (**right** panel). Subgroup analyses were performed corresponding to gender and HDL-C plasma concentration. Results are adjusted for age, gender, use of statins, CAD, BMI, diabetes mellitus, glycosylated hemoglobin, smoking, LDL/HDL cholesterol, triglycerides, and ApoAI.

Genetic associations with cholesterol efflux capacity were confirmed in subsequent analyses where we found a strong association between cholesterol efflux capacity and eGFR. Notably, even in patients with moderately reduced renal function, cholesterol efflux capacity was reduced as compared to those with normal renal function (Figure 2, left panel). These results were confirmed using serum uromodulin, which has recently been shown to be a useful marker for cardiovascular and renal health (Figure 2, right panel) [10]. In subgroup analyses, we showed that the association between eGFR and cholesterol efflux was stronger in men, whereas the association between serum uromodulin and efflux was much stronger in women. The subgroup analysis regarding plasma HDL-C levels showed the expected overall lower efflux values in patients with HDL levels below 35 mg/dl when compared to those with levels exceeding 35 mg/dl. However, grouping of patients by HDL did not affect the pattern of associations between cholesterol efflux with eGFR and uromodulin, respectively.

### 3.3. Cholesterol Efflux Capacity, Kidney Function, and Mortality

Among the 2468 persons studied, 717 deaths (29.2%) occurred during a median follow-up of 9.9 years. No associations were found between cholesterol efflux capacity and total mortality in adjusted models (Table 2, all-cause mortality, models 2 and 3). However, in patients within the lowest quartile of cholesterol efflux, the multivariable-adjusted risk for cardiovascular death was significantly increased (Table 2, cardiovascular mortality, model 2). The prognostic value of cholesterol efflux capacity was also seen after further adjustment for the intake of lipid-lowering drugs, cardiovascular risk factors, CAD status, and diabetes mellitus (Table 2, cardiovascular mortality, model 3).

Interestingly, inclusion of eGFR into the respective models did not reveal a major impact on the association between cholesterol efflux capacity and cardiovascular mortality (Table 2, cardiovascular mortality, model 4). The same was observed when we included genetic variants related to eGFR (Table 2, cardiovascular mortality, model 5).

## 4. Discussion

In this study, we analyzed cholesterol efflux capacity as one of the major HDL functions in patients who underwent coronary angiography. Patients were followed for a median of 9.9 years. We found a strong association between cholesterol efflux capacity and kidney function in our study population. Cholesterol efflux capacity was associated with cardiovascular mortality. A genetic score based on 53 SNPs related to eGFR, as well as the uromodulin-related polymorphism, rs12917707, were shown to be associated with cholesterol efflux capacity, but these genetic variants did not relate to CVD mortality. Since, in addition, cholesterol efflux capacity adjusted for eGFR or uromodulin was invariably associated with CVD mortality, we conclude that cholesterol efflux is not a mediator of adverse cardiovascular outcomes in renal insufficiency.

Patients with chronic kidney disease are at dramatically increased risk for CAD, which is accompanied by HDL dysfunction, including decreased cholesterol efflux capacity as early as in childhood and progressing in parallel to the loss of renal function [8,18,19,20]. In line with this, we observed that cholesterol efflux capacity was lower in moderate renal insufficiency patients and even more decreased in patients with severe renal insufficiency.

The association between cholesterol efflux capacity and cardiovascular mortality was only slightly weakened by inclusion of eGFR within the respective models, indicating that there is no direct link between cholesterol efflux and kidney function. Furthermore, genetic variants associated with eGFR or uromodulin were associated with cholesterol efflux, but not with CVD mortality. Taken together, cholesterol efflux and kidney function are modulating cardiovascular risk by different and independent mechanisms.

A limitation of our study is that the measurement of cholesterol efflux capacity does not reflect the whole of reverse cholesterol transport. However, cholesterol efflux is the first major step within this pathway and the technology used has been shown to be useful in several clinical studies by us and by others [15,21,22]. Additionally, our study group consisted of patients referred to coronary angiography. Therefore, our findings cannot be translated to the general population. Finally, lipoprotein parameters and cholesterol efflux capacity were measured once at baseline. We were not able to adjust for possible moderate fluctuations of these parameters during the follow-up.

Major strengths of this work are the detailed clinical and metabolic characterization of the LURIC participants and the long duration of the follow-up, with a large number of fatal cardiovascular events. Another advantage was the availability of genetic data. Accordingly, we were able to show the relationship between cholesterol efflux and kidney function by analyzing the association between efflux capacity and SNPs, which have been shown to be associated with eGFR in a meta-analysis of genome-wide association studies combining data from 133,413 individuals [16].

## 5. Conclusions

Our study supports the concept of dysfunctional HDL in chronic kidney disease patients. We found a strong association of cholesterol efflux capacity with kidney function as well as with cardiovascular mortality. However, detailed analyses of our data contradict the assumption that cholesterol efflux is mediating the effect of kidney function on cardiovascular outcomes. We are therefore proposing the view that cholesterol efflux and kidney function are exerting their effects on cardiovascular mortality via different and independent pathways.

## Figures and Tables

**Table 1:** Baseline characteristics of LURIC study participants.

	All	eGFR (mL/min per 1.73 m^2^)			
	(*n* = 2468)	>90 (*n* = 883)	60–90 (*n* = 1249)	≤60 (*n* = 331)	*p* *
Age (years, mean ± SD)	62.8 ± 10.4	56.0 ± 9.9	65.5 ± 8.3	71.1 ± 8.1	<0.001
Male sex (%)	68.1	78.0	64.8	54.1	<0.001
Body mass index (kg/m², mean ± SD)	27.5 ± 4.1	27.3 ± 4.0	27.7 ± 4.1	27.5 ± 4.4	0.002
Waist hip ratio (mean ± SD)	0.96 ± 0.08	0.96 ± 0.08	0.96 ± 0.08	0.96 ± 0.08	0.463
Systolic blood pressure (mmHg, mean ± SD)	141 ± 24	136 ± 22	143 ± 24	145 ± 24	0.081
Diastolic blood pressure (mmHg, mean ± SD)	81 ± 11	82 ± 11	82 ± 11	79 ± 12	0.001
Total cholesterol (mg/dL, mean ± SD)	208 ± 44	209 ± 46	209 ± 43	204 ± 48	0.175 ^‡^
LDL cholesterol (mg/dL, mean ± SD)	116 ± 35	116 ± 36	117 ± 34	110 ± 36	0.001 ^‡^
HDL cholesterol (mg/dL, mean ± SD)	39.1 ± 10.7	39.4 ± 10.5	39.6 ± 10.8	36.8 ± 10.9	<0.001 ^‡^
Effective HDL (mg/dL, mean ± SD)	35.1 ± 17.8	38.5 ± 18.1	34.9 ± 17.7	26.3 ± 13.9	<0.001 ^‡^
Apolipoprotein AI (mg/dL, mean ± SD)	130 ± 25	131 ± 25	131 ± 25	125 ± 26	<0.001 ^‡^
Apolipoprotein AII (mg/dL, mean ± SD)	41.8 ± 9.5	44.1 ± 9.3	41.3± 9.3	37.4 ± 9.3	<0.001 ^‡^
Apolipoprotein B (mg/dL, mean ± SD)	104 ± 25	104 ± 26	104 ± 24	103 ± 27	0.491 ^‡^
Triglycerides (mg/dL, median, Q1 to Q3)	146 (108–201)	144 (106–199)	143 (107–197)	156 (119–214)	<0.001 ^‡,§^
CETP (μg/mL, median, Q1 to Q3)	1.12 (0.86–1.49)	1.10 (0.84–1.45)	1.13 (0.87–1.52)	1.16 (0.86–1.57)	0.048 ^§^
CRP (mg/dL, mean ± SD)	1.72 ± 0.77	1.56 ± 0.72	1.74 ± 0.78	2.06 ± 0.78	<0.001
SAA (mg/L, mean ± SD)	29.8 ± 114.7	22.6 ± 90.8	28.8 ± 106.8	52.8 ± 179.7	0.002
Urea (mg/dL, mean ± SD)	39.3 ± 15.2	32.4 ± 8.2	38.5 ± 9.8	60.2 ± 23.5	<0.001
Diabetes mellitus (%)	28.9	20.8	30.7	43.5	<0.001
Lipid lowering therapy (%)	49.7	49.9	49.6	49.8	0.990
CAD (%)	77.0	73.0	78.4	82.5	0.001
Smoking					
Never (%)	37.5	29.4	40.8	46.5	
Past (%)	43.2	43.4	42.9	44.1	
Current (%)	19.3	27.2	16.3	9.4	<0.001
eGFR (ml/min per 1.73 m^2^)	81.6 ± 19.9	101.4 ± 7.9	77.0 ± 8.4	46.2 ± 11.2	<0.001

* Analysis of variance or logistic regression, respectively, adjusted for age and gender. ^‡^ Adjusted for use of lipid lowering drugs. ^§^ ANOVA of logarithmically transformed values.

**Table 2:** Hazard ratio for cardiovascular death according to cholesterol efflux.

Cardiovascular Mortality						
	**Model 1 HR**		**Model 2 HR**		**Model 3 HR**	
	**(95% CI)**	***p***	**(95% CI)**	***p***	**(95% CI)**	***p***
**Efflux**						
**Quartile**						
**1st**	1.0^ref^		1.0^ref^		1.0^ref^	
**2nd**	**0.756 (0.590–0.968)**	**0.026**	0.800 (0.625–1.025)	0.077	0.814 (0.634–1.044)	0.105
**3rd**	**0.658 (0.509–0.852)**	**0.001**	**0.711 (0.549–0.920)**	**0.010**	**0.723 (0.557–0.939)**	**0.015**
**4th**	**0.632 (0.488–0.818)**	**0.001**	**0.701 (0.540–0.910)**	**0.008**	**0.758 (0.582–0.988)**	**0.040**
	**Model 4 HR**		**Model 5 HR**			
	**(95% CI)**	***p***	**(95% CI)**	***p***		
**Efflux**						
**Quartile**						
**1st**	1.0^ref^		1.0^ref^			
**2nd**	0.820 (0.639–1.052)	0.118	0.813 (0.623–1.061)	0.127		
**3rd**	**0.722 (0.556–0.938)**	**0.015**	**0.725 (0.548–0.959)**	**0.024**		
**4th**	0.793 (0.608–1.035)	0.087	0.765 (0.577–1.013)	0.062		

Model 1: not adjusted. Model 2: adjusted for age and gender. Model 3: adjusted for age, gender, use of statins, CAD, BMI, diabetes mellitus, smoking, CRP, LDL/HDL cholesterol, and triglycerides. Model 4: model 3 + adjustment for eGFR. Model 5: model 3 + adjustment for score53 (eGFR related SNPs). CI = confidence interval, HR = hazard ratio. Significant results are presented in bold.

## Data Availability

The datasets used and/or analyzed during the current study are available from the corresponding author on reasonable request.
